# Evaluating the impact of larviciding with *Bti* and community education and mobilization as supplementary integrated vector management interventions for malaria control in Kenya and Ethiopia

**DOI:** 10.1186/s12936-020-03464-6

**Published:** 2020-11-03

**Authors:** Clifford M. Mutero, Collins Okoyo, Melaku Girma, Joseph Mwangangi, Lydia Kibe, Peter Ng’ang’a, Dereje Kussa, Gracious Diiro, Hippolyte Affognon, Charles M. Mbogo

**Affiliations:** 1 International Centre of Insect Physiology and Ecology (ICIPE), P. O. Box 30772, 00100 Nairobi, Kenya; 2 Institute for Sustainable Malaria Control, School of Health Systems and Public Health, University of Pretoria, Pretoria, South Africa; 3 Eastern and Southern Africa Centre of International Parasite Control, Kenya Medical Research Institute (KEMRI), Nairobi, Kenya; 4 Zoological Sciences Department, Addis Ababa University, Addis Ababa, Ethiopia; 5 Centre for Geographic Medicine Research, Kenya Medical Research Institute (KEMRI), Kilifi, Kenya; 6 International Centre of Insect Physiology and Ecology (ICIPE), Addis Ababa, Ethiopia; 7 West and Central Africa Council for Agricultural Research and Development, Dakar, Senegal

**Keywords:** Malaria, Integrated vector management, Community education and mobilization, Larviciding, *Bti*

## Abstract

**Background:**

Malaria prevention in Africa is mainly through the use of long-lasting insecticide treated nets (LLINs). The objective of the study was to assess the effect of supplementing LLINs with either larviciding with *Bacillus thuringiensis israelensis (Bti)* or community education and mobilization (CEM), or with both interventions in the context of integrated vector management (IVM).

**Methods:**

The study involved a factorial, cluster-randomized, controlled trial conducted in Malindi and Nyabondo sites in Kenya and Tolay site in Ethiopia, to assess the impact of the following four intervention options on mosquitoes and malaria prevalence: LLINs only (arm 1); LLINs and *Bti* (arm 2); LLINs and CEM (arm 3); and, LLINs combined with *Bti* and CEM (arm 4). Between January 2013 and December 2015, CDC light traps were used to sample adult mosquitoes during the second, third and fourth quarter of each year in 10 houses in each of 16 villages at each of the three study sites. Larvae were sampled once a fortnight from potential mosquito-breeding habitats using standard plastic dippers. Cross-sectional malaria parasite prevalence surveys were conducted involving a total of 11,846 primary school children during the 3-year period, including 4800 children in Tolay, 3000 in Malindi and 4046 in Nyabondo study sites.

**Results:**

Baseline relative indoor anopheline density was 0.11, 0.05 and 0.02 mosquitoes per house per night in Malindi, Tolay and Nyabondo sites, respectively. Nyabondo had the highest recorded overall average malaria prevalence among school children at 32.4%, followed by Malindi with 5.7% and Tolay 1.7%. There was no significant reduction in adult anopheline density at each of the three sites, which could be attributed to adding of the supplementary interventions to the usage of LLINs. Malaria prevalence was significantly reduced by 50% in Tolay when using LLINs coupled with application of *Bti,* community education and mobilization. The two other sites did not reveal significant reduction of prevalence as a result of combining LLINs with any of the other supplementary interventions.

**Conclusion:**

Combining LLINs with larviciding with *Bti* and CEM further reduced malaria infection in a low prevalence setting in Ethiopia, but not at sites with relatively higher prevalence in Kenya. More research is necessary at the selected sites in Kenya to periodically determine the suite of vector control interventions and broader disease management strategies, which when integrated would further reduce adult anopheline populations and malaria prevalence beyond what is achieved with LLINs.

## Background

After more than two decades of malaria being associated with an estimated one million global deaths annually prior to 2000, the disease cases declined by 40–60% in many countries between 2000 and 2015 [1]. Malaria deaths stood at 435,000 globally in 2017, far below the 2000 levels despite a slowdown in the rate of decline reported from 2015 onwards [2]. The decrease in malaria burden in nearly all the endemic countries since 2000 is commonly attributed to a multi-pronged strategy involving the scaling up of the use of long-lasting insecticide-treated nets (LLINs), selective indoor residual spraying (IRS) and better access to prompt diagnosis and effective treatment of the disease [3–5], among other programmatic interventions. Other factors that have been speculated as generally having contributed to a decline of malaria vectors and malaria itself in endemic countries include climatic and environmental changes and general development leading to better socio-economic conditions including improved housing [6–8].

Unfortunately, as different countries pursue the goal of malaria elimination, encouraged by the gains experienced since 2000, serious concerns have been expressed at national and international level regarding sustainability of the primary interventions, particularly those targeting vector control. In the first instance, there has been a looming threat posed to malaria control programmes by widespread vector resistance to the insecticides commonly used in IRS and LLINs [9, 10]. Secondly, the ability of governments in many malaria-affected countries to finance prevention and control activities outside the ambit of external donor-funded programmes is often in doubt [11]. In recognition of these, among other technical, community and health system challenges, the World Health Organization (WHO) has repeatedly recommended the adoption of integrated vector management (IVM) among the strategies that could lead to sustainable malaria control and, ultimately, its elimination [12, 13]. Key elements of IVM include evidence-based decision making, the integration of non-chemical and chemical vector control methods, advocacy and social mobilization, and inter-sectoral collaboration and action. The key elements have recently been re-emphasised and endorsed by the World Health Assembly (WHA) in the form of pillars of action in the Global Vector Control Response framework for 2017–2030 [14].

However, research evidence is largely unavailable to support the perception of greater impact of IVM on malaria when compared to relatively non-integrated vector control in different eco-epidemiological settings. Towards this end, several studies have been conducted to assess whether adding a secondary anti-vector intervention in a setting where the primary vector control method is the usage of LLINs can lead to further site-specific decline of malaria prevalence towards elimination. The secondary methods most generally evaluated include IRS, screening of houses with fine wire mesh to reduce mosquito entry and consequently reducing vector-host contact, and larval source management, particularly involving biolarviciding with *Bacillus thuringiensis israelensis* (*Bti*) [15–18]. The relevance and urgency of such research has received new impetus following a stalling in the general decline of malaria noted from around 2015 [2].

IVM for malaria control has previously been promoted in areas where the present studies were conducted in Kenya and Ethiopia through collaborative research and non-research developmental activities [19]. The IVM strategy adopted in those previous studies involved community education and mobilization geared towards improved environmental management by the local community and other stakeholders, routine application of the common biopesticide *Bti* in potential mosquito breeding sites, and the promotion of proper use of LLINs [20–24]. An external review conducted in 2012 to evaluate this earlier work noted that while participatory IVM may have led to significant reductions in malaria in the study sites, it was nevertheless not possible to tease out and quantify the incremental effects of the IVM effort, over and above those ordinarily due to the conventional singular usage of LLINs [19, 25].

The objective of this study was therefore to determine the impact of adding larviciding using *Bti* and community education and mobilization (CEM) onto the frontline intervention of LLINs. It specifically aimed at verifying under field conditions if adding the former two interventions, herein, also referred to as supplementary interventions, either individually or combined can further reduce indoor malaria vector density and malaria prevalence in the community compared to when LLINs alone are used.

## Methods

### Study areas and context

**Fig. 1:**
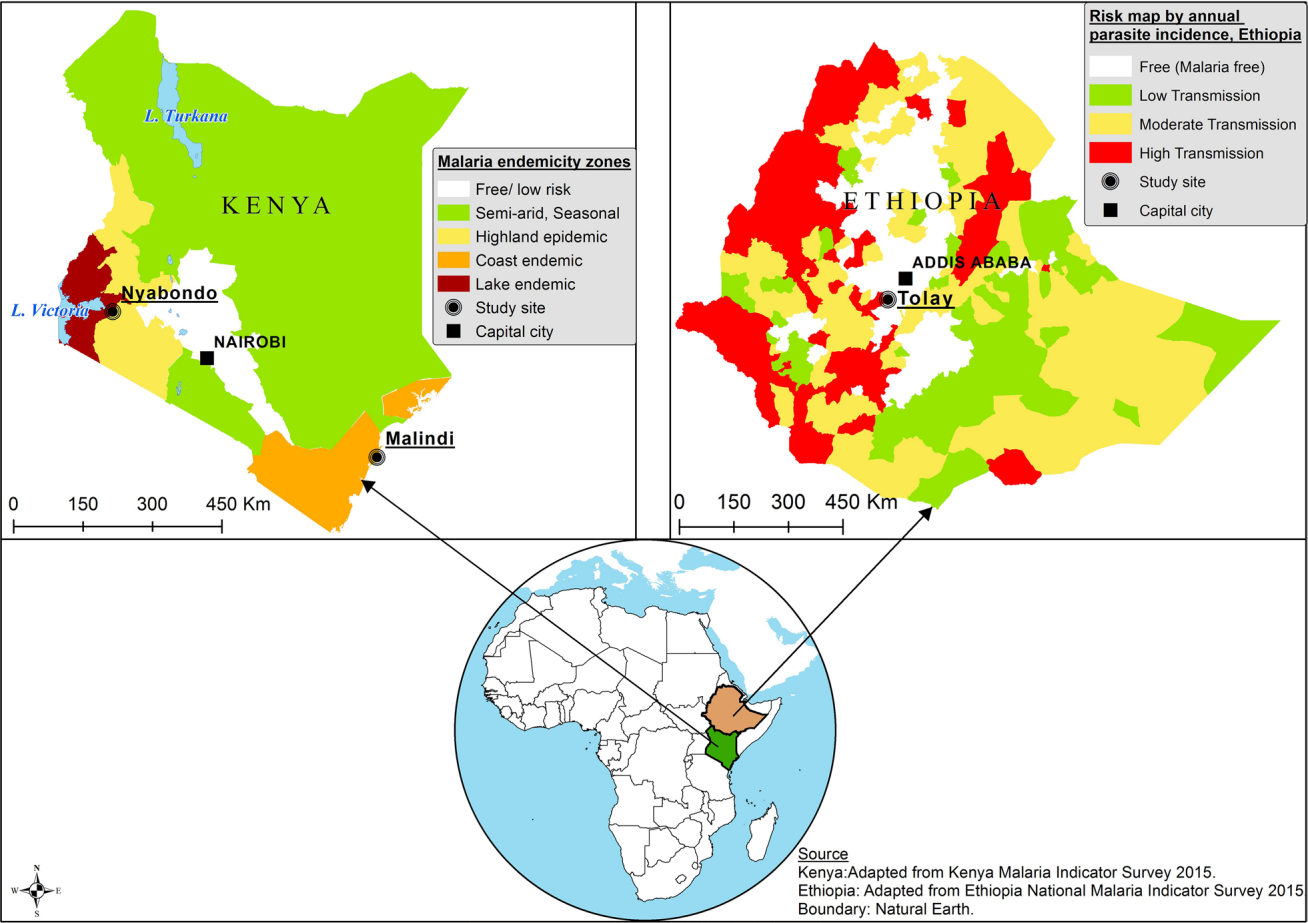
Locational map of the study sites

The studies were conducted from January 2013 to December 2015 simultaneously in three different geographic locations: the Nyabondo plateau in the western part of Kenya, Malindi Sub-County at the Kenyan coast, and the Tolay region in southwestern Ethiopia (Fig. 1). The three study sites were selected to enable comparisons of the likely effectiveness and impacts of IVM to be made between situations of low and moderate to high malaria prevalence, represented, respectively, by Tolay on the one hand, and Malindi and Nyabondo on the other [3, 4].

Nyabondo is a rural setting in a plateau located in Upper Nyakach Division of Kisumu County (0° 23′ S; 34° 58′ E), about 30 km northeast of Lake Victoria, at an altitude between 1520 and 1670 m above sea level. An estimated 34,000 people live in homesteads spread out in the area but administratively grouped into villages, each of up to about 200 houses. The main livelihood activities include subsistence farming, mainly of maize, small-scale livestock rearing and brick-making. Water accumulation in shallow ground pits from the brick-making activity contributes to mosquito breeding [20]. Other breeding sites commonly recorded in Nyabondo include abandoned and poorly managed fish ponds [26]. Malaria is endemic in the Lake Victoria region, with a reported average prevalence of 27% in 2015 [3]. This is the highest average parasite rate among the different malaria-endemic regions in Kenya. Malaria peak seasons follow the long and short rains but this may vary from year to year. Vectors of malaria in Nyabondo mainly belong to the species *Anopheles arabiensis* [20]. Local houses are constructed of mud walls, iron sheet roofs and have narrow open eaves [27].

Malindi Sub-County is located in Kenya’s coastal area about 108 km north of Mombasa (3° 13′ S; 40° 7′ E) at an altitude of between 40 and 400 m above sea level. This study was conducted in a rural area of Malindi with approximately 50,000 people. Subsistence farming, fishing and trading are the major economic activities in this area. Malaria is one of the key causes of morbidity and mortality within the Sub-County with an average prevalence of 4–8% [3]. The main vectors of malaria in Malindi are *An. arabiensis, Anopheles merus* and *Anopheles funestus* [8]. There are two main rainy seasons in the Kenyan study areas, i.e., the long (March to June) and short (September to November) rains. Common mosquito breeding sites include shallow ponds, drainage channels and wells for domestic water use. Houses in the study area are constructed of mud walls, palm thatched roofs and have wide open eaves.

Tolay is a rural semi-arid, agro-climatic zone located in the southwestern part of Ethiopia (8° 14′ N; 37° 35′ E) at an altitude of 1100–1600 m above sea level. The community is mainly engaged in small-scale mixed agriculture and trade at village level. The major food crops in the region are maize, sorghum and teff. Malaria is unstable and seasonal, with a prevalence of about 2% or less [4]. Peak malaria transmission and epidemic season includes the period from October to November, coinciding with the peak rain season. The main vector of malaria in Tolay is *An. arabiensis* [21]. Common mosquito-breeding sites include semi-permanent and permanent ponds, pools at the edge of streams and drainage ditches. Most houses in Tolay are constructed of wooden walls plastered with mud and cow dung, and grass thatch or corrugated iron sheet roofs. The houses do not have open eaves.

### Study interventions

#### LLINs (experimental control)

All the three project sites were in areas with universal coverage with LLINs, distributed by the countries’ national malaria control programmes (NMCPs) via multiple channels, including free mass and continuous distributions mainly through antenatal and immunization services [3, 4, 28]. Mass distribution of LLINs had been conducted in the respective study sites in Kenya and Ethiopia in 2012 [3, 4]. Universal coverage implies that people at risk of malaria own LLINs at a ratio of one net for every two persons sleeping in a household. Thus, the existing situation of LLIN coverage constituted the default experimental control treatment to which one other mosquito control (larviciding with *Bti*) and one social intervention (community education and mobilization) were added either individually or in combination. Survey information available retrospectively regarding household LLIN ownership at the beginning of the baseline data collection in 2013 indicates household net ownership of 83% in Nyabondo area, 84% in Malindi and 64% in Tolay [4, 23, 29].

#### Larviciding with *Bti*

Application of *Bti* granules (VectoBac G; Valent Biosciences Corp, Libertyville, IL, USA) was done once every 2 weeks to all potential *Anopheles* breeding habitats, identified in advance at a particular study site where this intervention was to be implemented. All habitats found to have anopheline mosquito larvae within a study village were treated. Also included were those habitats within a 200-m buffer zone from the last house in the village in order to mitigate potential spillover effects in entomological assessments due to mosquito flight range [30]. The *Bti* was applied by hand, by a team of 6–8 people, half of whom were project field staff and the other half members of the community, commonly referred to as mosquito scouts [19, 25], who had been trained locally by the project staff on how to apply the product. The *Bti* granules were broadcast by hand within the manufacturer’s recommended dose of about 3.0 kg/ha [31].

#### Community education and mobilization

The community education and mobilization intervention entailed conducting intensive community educational activities deliberately planned to surpass the awareness creation that normally accompanies LLIN distribution by the Ministry of Health (MOH) of either country. In all three project sites, resident project field staff of about 2–3 persons conducted the CEM activities on regular monthly basis assisted by about 6–10 mosquito scouts. The scouts had previously been trained by the research team in basic aspects of IVM including: visually identifying presence of mosquito larvae in aquatic habitats, larval source management like draining of stagnant water and filling up of temporary pools of water with soil or levelling them wherever appropriate, proper care and usage of mosquito nets, adult mosquito identification, and community participation in malaria prevention and control. The following methods of community education and mobilization were applied: door to door campaigns, engagement of primary school pupils through school health and environment clubs, distribution of information, education and communication (IEC) materials, neighbourhood clean-up campaigns with emphasis on elimination of mosquito breeding and resting habitats, and community meetings on mosquito and malaria control activities.

### Study design

**Fig. 2:**
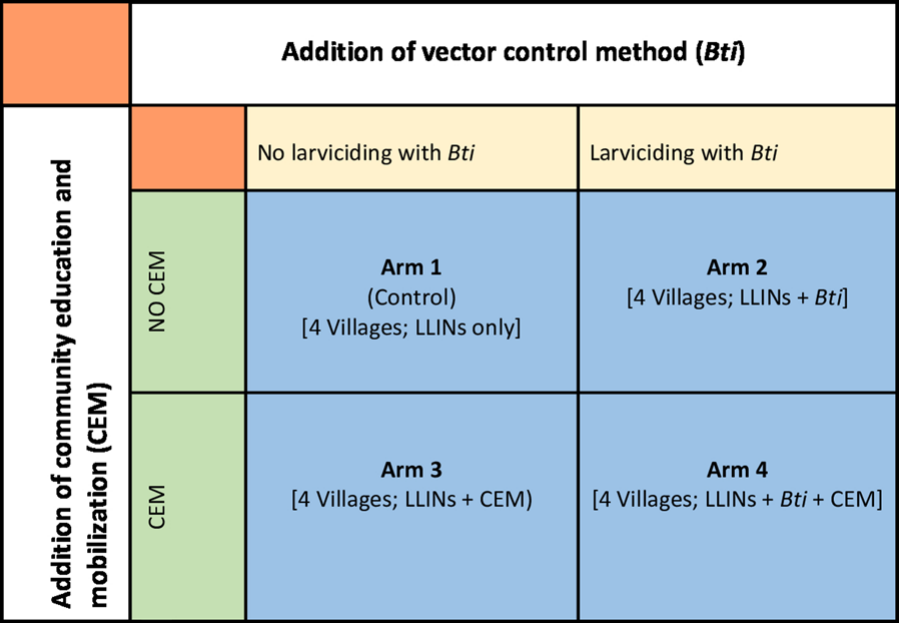
Factorial study design

A cluster-randomized, controlled trial with a factorial design [32, 33] was implemented between January 2013 and December 2015 to study the effects of four intervention treatments (arms) at each of the three study sites (Fig. 2). In each site there were 16 villages (clusters), with four villages randomly assigned to each of the four study arms: Arm 1 comprised LLIN use only; Arm 2 comprised LLIN use and application of *Bti*; Arm 3 comprised LLIN use and community education and mobilization; and, Arm 4 comprised LLIN use, application of *Bti* and community education and mobilization. In each village at each site, 10 houses were randomly included in entomological assessment using indoor adult anopheline mosquito relative density as the proxy indicator of malaria transmission risk. A total of 38 schools located within the study sites (10 schools in Malindi, 12 in Nyabondo, 16 in Tolay) were randomly sampled for cross-sectional malaria parasitological surveys among school children, using parasite prevalence as the measure of epidemiological impact. The overriding hypothesis was that integrating usage of LLINs with CEM and larviciding with *Bti* (arm 4) has greater impact in reducing populations of anopheline mosquitoes and malaria prevalence than when LLINs are used alone or integrated with only either one of the other two supplementary interventions.

The study villages at each study site were randomly selected from a larger pool of villages, which were purposively chosen from the entire list of villages in each study site, and fulfilled most or all of the following criteria: ecologically similar including, among other features, the presence of natural or man-made potential mosquito breeding habitats; location in an area with reported universal MOH-LLIN coverage; accessibility throughout the year; availability of a local primary school; existence of a local health facility such as dispensary or hospital. During selection of the 16 villages, a minimum distance of approximately 0.5 km was maintained between study households in one village and those in the next in order to mitigate potential spillover of intervention effects [30, 34].

### Larval mosquito sampling

Mosquito larvae were sampled once every 2 weeks from all the study villages. All stagnant water bodies within and about 200 m from the last house in a village were inspected for mosquito larvae using a standard dipper (11.5 cm diameter and 350 ml capacity) [35]. Larval sampling was carried out between 09:00 and 12:00 by mosquito scouts from the study villages and trained by the research team. The number of scoops varied according to the size of micro-habitat and ranged from one to 10 scoops per habitat for small and large habitats, respectively. The collected larvae were identified as either anopheline or culicine genera using morphological identification keys [36] and their number recorded. Information on mosquito breeding habitat types was recorded.

### Adult mosquito sampling

Adult mosquitoes were sampled indoors once every 3 months from a total of 160 houses at each of the three project sites. The total number of houses comprised of 10 houses from each of the 16 villages, initially selected using simple random sampling. These same houses continued to be used for the rest of the study for ease of field logistics. Consent of the house owners was sought in order to make the houses accessible for the duration of the study. Mosquitoes were collected indoors using standard Centers for Disease Control (CDC) light traps (Model 512; John W. Hock Co, Gainesville, FL, USA). In each selected house, the trap was hung about 1.5 m from the floor, at the rear end of a regularly used bed, with the occupant protected with a bed net [37]. Mosquito scouts deployed the traps at 18:00 and collected them the following morning at 06:00. Mosquitoes collected were sorted in the field laboratory and morphologically identified as belonging to either anopheline or culicine genera. Anophelines were further identified to distinguish between the main malaria vector, *Anopheles gambiae* sensu lato (*s.l*.) species complex and other *Anopheles* species [36]. Finally, sub-samples of *An. gambiae s.l.* were analysed for sibling species composition using recombinant deoxyribonucleic acid (rDNA)-polymerase chain reaction (PCR) technique [38–40]. The relative density of mosquitoes in a village was calculated by dividing the total number collected by the number of houses sampled in order to obtain the average number per house per night.

### Cross-sectional malaria surveys

Cross-sectional malaria parasite surveys in children aged between 6 and 12 years were conducted at each project site to assess the local prevalence of malaria. A total of 38 public primary schools were used in the study, including 12 schools in Nyabondo, 10 in Malindi and 16 in Tolay. The schools were intended to be matched with the study villages at a ratio of one school per village. However, this was possible in Tolay but not in Malindi and Nyabondo. For this reason, some of the study arms at the latter two sites had fewer than four schools included in the prevalence assessments in spite of them having four study villages. In each school, 20 children of equal number of males and females were randomly selected from each of classes 2–6 based on the children present that day, and using computer-generated random number tables. This sample size of 100 children per school sought to estimate a 5% change in prevalence of infection across the years, assuming a power of 80% and test size of 5%. Oversampling of 20% was considered to take care of any contingencies such as drop-outs, data entry error, non-response, and damaged samples.

*Plasmodium falciparum* prevalence (*Pf*PR) [41] was established through rapid diagnostic tests (RDT, Paracheck Ver.3, Orchid Biomedical Systems, Mumbai, India). The selected children were asked to provide a finger-prick blood sample, which was used to assess *Plasmodium* infection in the peripheral blood. The sample collection was done by trained MOH technicians. All children who were found to be positive were treated with artemisinin-based combination therapy (ACT), artemether-lumefantrine, by the MOH staff according to the national guidelines for the diagnosis, treatment and prevention of malaria in Kenya and Ethiopia [3, 4].

### Statistical analysis

The primary objective of the statistical analysis was to: (1) determine the malaria transmission risk as measured by the relative vector density each year of the survey; (2) determine the prevalence of *P. falciparum* infection in school children as assessed by the school-level cross-sectional surveys; and, (3) estimate the magnitude of the intervention effects over time for the four intervention groups on malaria transmission risk and *P. falciparum* infection prevalence.

#### Entomological analysis for vector characteristics and treatment effects

The following key entomological indicators for measuring vector characteristics were calculated: (1) adult mosquito relative density defined as the number of adult mosquitoes per house per night; and (2) mosquito larval density defined as the number of mosquito larvae per dip. Vector density was estimated using negative binomial regression and adjusted for the number of habitats (for larval mosquitoes) and household clusters (for adult mosquitoes) with the number of trap-nights included as an offset to account for differences in collection effort in the mosquito collection method. The effect of the treatment interventions was estimated using generalized estimating equations (GEE) on vector density, allowing for within-subject correlation using robust variance estimator to calculate standard errors (SEs). From the GEE model, the incidence rate ratios (IRRs) were reported and herein described as density ratios (DR), the control intervention (LLINs only) was used as the reference against the other interventions.

#### *Plasmodium falciparum* prevalence and treatment effects

*Plasmodium falciparum* infection was defined as a positive RDT result [41–43]. Proportions were calculated for variables of interest at school level with a 95% confidence interval (CI) using generalized linear models that accounted for school clusters. Overall, analysis of the treatment effect of the various interventions on infection prevalence was initially analysed by fitting GEE model assuming within-subject correlation and binomial family function to test whether the proportion positive for *P. falciparum* infection varied significantly among the treatment arms. The covariates included in the model were treatment interventions, age, gender, and study site. Additionally, multivariable mixed effects logistic regression model at three levels was separately fitted, individuals nested within schools selected within treatment groups and finally within study site with age and gender retained as fixed terms in the model and reporting the adjusted odds ratio (aOR). The results from the first and second model were compared and found no significant difference between the two models. Hence, the results of the latter model only were reported here.

All statistical analyses were performed using STATA version 14.1 (STATA Corporation, College Station, TX, USA). Graphs were developed using the *ggplot* package implemented in R [44].

## Results

### Entomological findings from adult and larval mosquito collection

Table 1 provides the baseline household and entomological characteristics. The overall baseline mean number of adult anopheline (χ^2^ = 101.85, p = 0.090) and culicine (χ^2^ = 146.84, p = 0.178) mosquito densities did not vary significantly in the treatment intervention groups. Similarly, the baseline larval anopheline and culicine mosquito densities did not vary significantly in the treatment intervention groups.

**Table 1:** Baseline characteristics

Characteristics	Overall	Arm 1	Arm 2	Arm 3	Arm 4
Villages per site (houses per village)	16 (10)	4 (10)	4 (10)	4 (10)	4 (10)
Household membership: median number (range; N)^a^					
Malindi	8 (2–22; 1999)	8 (2–18; 999)	8 (3–19; 600)	7 (3–18; 200)	9 (2–22; 200)
Nyabondo	4 (1–9; 1546)	5 (1–8; 365)	4 (1–8; 388)	4 (1–8; 374)	4 (1–9; 419)
Tolay	5 (1–10; 3862)	5 (1–10; 967)	6 (3–10; 1109)	5 (2–9; 958)	4 (1–10; 828)
Overall	5 (1–22; 7407)	5 (1–18; 2331)	6 (1–19; 2097)	5 (1–18; 1532)	4 (1–22; 1447)
Children characteristics^c^					
Median age, years (range; N)					
Malindi	7 (3–15; 1000)	7 (5–13; 500)	7 (6–13; 300)	7 (3–10; 100)	9 (3–15; 100)
Nyabondo	8 (4–13; 999)	9 (6–13; 200)	9 (4–13; 212)	8 (5–13; 145)	9 (5–13; 153)
Tolay	12 (4–19; 1600)	12 (4–19; 400)	11 (7–17; 400)	12 (6–16; 400)	12 (5–17; 400)
Overall	9 (3–19; 3599)	9 (4–19; 1100)	9 (4–17; 912)	10 (3–16; 645)	11 (3–17; 653)
Baseline malaria infection prevalence %(95% CI)					
Malindi	5.4 (2.4–12.1)	1.6 (0.6–4.0)	12.0 (4.4–32.6)	4.0 (1.5–10.4)	6.0 (2.8–13.0)
Nyabondo	24.1 (17.0–34.1)	24.5 (12.1–49.7)	34.9 (25.9–47.0)	17.9 (12.0–26.8)	25.5 (19.6–33.2)
Tolay	1.4 (0.9–2.2)	2.3 (1.5–3.4)	1.3 (0.3–5.5)	0.8 (0.4–1.4)	1.3 (0.5–3.4)
Overall	8.8 (5.5–14.0)	6.0 (2.7–13.3)	12.6 (6.6–24.2)	5.1 (2.3–11.4)	7.7 (3.6–16.2)
Entomological characteristics^d^					
Baseline mean number of vectors per house per night %(95% CI)					
*Anopheles* spp.^b^					
Malindi	0.11 (0.07–17.9)	0.18 (0.08–0.39)	0.09 (0.03–0.26)	0.04 (0.01–0.19)	0.14 (0.06–0.32)
Nyabondo	0.02 (0.01–0.05)	0 (0–0.14)	0.04 (0.01–0.13)	0.04 (0.01–0.13)	0.02 (0–0.05)
Tolay	0.05 (0.02–0.09)	0.05 (0.01–0.20)	0.06 (0.01–0.21)	0.06 (0.02–0.21)	0.03 (0.01–0.18)
Overall	0.05 (0.04–0.07)	0.06 (0.03–0.11)	0.06 (0.03–0.11)	0.04 (0.02–0.10)	0.05 (0.02–0.09)
*Culex* spp.^b^					
Malindi	0.77 (0.57–1.04)	2.24 (1.16–4.30)	0.29 (0.16–0.52)	0.57 (0.37–0.88)	0.20 (0.10–0.40)
Nyabondo	0.13 (0.10–0.18)	0.12 (0.06–0.24)	0.12 (0.06–0.23)	0.04 (0.01–0.14)	0.20 (0.13–0.33)
Tolay	0.06 (0.02–0.11)	0.06 (0.02–0.22)	0.04 (0.01–0.19)	0.04 (0.01–0.19)	0.08 (0.03–0.24)
Overall	0.26 (0.22–0.32)	0.59 (0.38–0.90)	0.14 (0.09–0.22)	0.19 (0.13–0.28)	0.32 (0.12–0.26)
Baseline mean number of larvae per dipper % (95% CI)					
*Anopheles* spp.					
Malindi	1.03 (0.43–2.42)	0	1.60 (0.64–4.02)	0.09 (0.01–1.30)	0
Nyabondo	0.11 (0.10–0.12)	0.12 (0.10–0.14)	0.08 (0.07–0.10)	0.11 (0.09–0.12)	0.13 (0.11–0.15)
Tolay	0.81 (0.67–0.97)	0.58 (0.39–0.87)	0.96 (0.72–1.28)	0.81 (0.54–1.22)	0.83 (0.57–1.20)
Overall	0.14 (0.13–0.14)	0.14 (0.12–0.16)	0.14 (0.12–0.15)	0.12 (0.10–0.13)	0.15 (0.13–0.17)
*Culex* spp.					
Malindi	0.53 (0.27–1.05)	0	0.71 (0.30–1.66)	0.57 (0.16–1.99)	0.16 (0.03–0.75)
Nyabondo	0.19 (0.18–0.20)	0.20 (0.17–0.22)	0.11 (0.10–0.13)	0.23 (0.21–0.25)	0.21 (0.19–0.24)
Tolay	1.16 (0.99–1.35)	1.37 (1.01–1.87)	0.99 (0.75–1.32)	1.15 (0.72–1.84)	1.14 (0.88–1.48)
Overall	0.22 (0.20–0.23)	0.23 (0.20–0.27)	0.15 (0.13–0.16)	0.25 (0.23–0.27)	0.25 (0.22–0.27)

^a^Reported household membership is cumulative of the three years since they were not collected during baseline

^b^Adult vector density was calculated for only those mosquitoes collected using CDC light trap technique

^c^Baseline malaria prevalence survey was done in October and November 2013 for Malindi site, January 2014 for Nyabondo site and May 2014 for Tolay site

^d^Baseline entomological survey was done in 2013 for all the three sites

Arm 1 referred to LLINs only; Arm 2 combination of LLINs and *Bti*; Arm 3 combination of LLINs and CEM; and Arm 4 combination of LLINs, *Bti*, and CEM

Overall, 24,763 adult anopheline and culicine mosquitoes were collected over the three years in all the three study sites with majority being culicines (83.4%, n = 20,640). The *Anopheles* captured included *An. gambiae* (14.1%, n = 3501), *An. funestus* (1.9%, n = 471), and other *Anopheles* species (0.6%, n = 151). Over half (69.8%, n = 14,398) of all the culicine mosquitoes collected were from Nyabondo site, followed by Malindi (28.4%, n = 5859) and Tolay (1.9%, n = 383). On the other hand, the majority of all the anopheline mosquitoes collected were from Malindi site (56.6%, n = 2334), followed by Nyabondo (37.5%, n = 1547) and Tolay (5.9%, n = 242).

*Anopheles arabiensis* was the predominant *An. gambiae* sibling species in all the sites being more than 99% in Tolay and Nyabondo where the number of specimens identified by PCR were 94 and 100, respectively. The composition of *An. gambiae s.l.* in Malindi as determined from 207 specimens identified by PCR was *An. arabiensis* (64%) and *Anopheles merus* (36%). The sibling species *An. gambiae* sensu stricto of the *An. gambiae* complex was virtually absent at the three project sites.

**Fig. 3:**
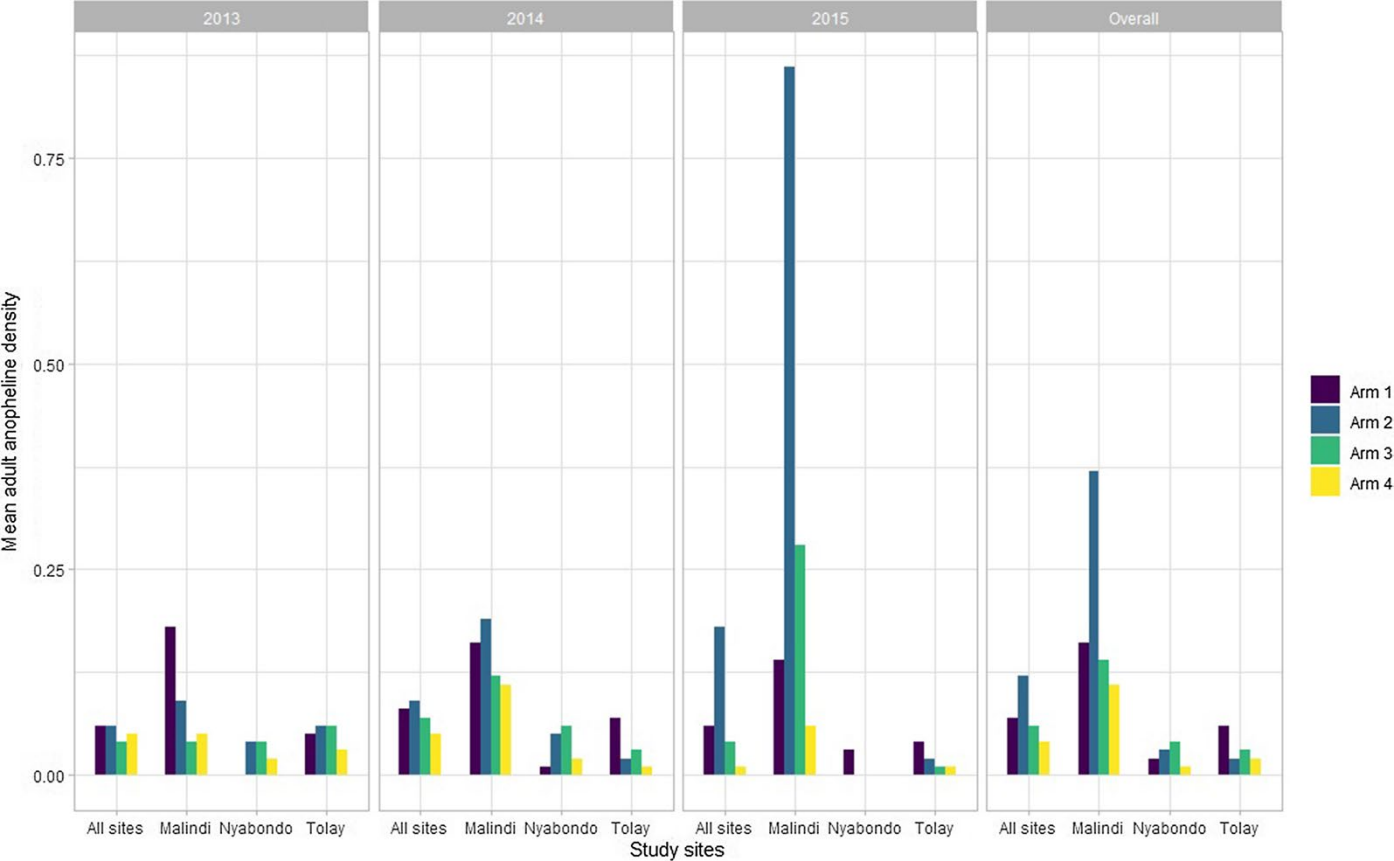
Mean adult mosquito densities at different survey years in Malindi, Nyabondo and Tolay sites

The overall adult anopheline mosquito density was 0.07 with site-specific anopheline density of 0.20, 0.03 and 0.02 for Malindi, Tolay and Nyabondo sites, respectively. The least adult anopheline density was observed in arm 4 (0.04) and arm 3 (0.06) of the study. Notably, arm 4 of the study showed least adult anopheline density in all the three sites (Fig. 3). Similarly, the overall adult culicine density was 0.14 with highest density observed in Malindi study site. Least adult culicines density was observed in arm 4 (0.08) and arm 3 (0.11) of the study. In two study sites, Malindi and Tolay, least adult culicines density was seen in arm 4 of the study while in Nyabondo site it was seen in arm 3.

Analysis of the effect of the treatment interventions on adult vector density showed that overall arm 4 of the study significantly reduced the adult anophelines density by nearly half (DR = 0.55, p = 0.012), and also significantly reduced adult culicines density by nearly two-thirds (DR = 0.38, p < 0.001). In all the three study sites, arm 4 non-significantly reduced both the adult anophelines and culicines density (Table 2).

**Table 2:** Comparison of the effects of the treatment interventions on adult mosquito density in the sampled villages in Malindi, Nyabondo and Tolay study sites using generalized estimating equations

Interventions	Overall	Year 1: 2013	Year 2: 2014	Year 3: 2015
	DR, p-value	DR, p-value	DR, p-value	DR, p-value
*Adult anopheline mosquito density*				
Overall				
Arm 1	Reference			
Arm 2	1.78 (1.25–2.55), p = 0.002	1.03 (0.39–2.70), p = 0.955	1.13 (0.66–1.96), p = 0.654	3.24 (1.79–5.85), p < 0.001
Arm 3	0.88 (0.58–1.34), p = 0.561	0.78 (0.27–2.24), p = 0.639	0.98 (0.56–1.72), p = 0.949	0.76 (0.35–1.66), p = 0.493
Arm 4	0.55 (0.34–0.88), p = 0.012	0.82 (0.31–2.13), p = 0.678	0.62 (0.33–1.19), p = 0.150	0.24 (0.07–0.77), p = 0.016
Malindi				
Arm 1	Reference			
Arm 2	2.36 (1.51–3.71), p < 0.001	0.51 (0.13–2.05), p = 0.346	1.19 (0.60–2.37), p = 0.611	6.17 (2.71–14.06), p < 0.001
Arm 3	0.86 (0.49–1.49), p = 0.582	0.24 (0.04–1.38), p = 0.109	0.77 (0.37–1.60), p = 0.479	2.00 (0.72–5.57), p = 0.182
Arm 4	0.69 (0.38–1.25), p = 0.224	0.79 (0.23–2.68), p = 0.704	0.69 (0.32–1.48), p = 0.342	0.42 (0.08–2.33), p = 0.320
Nyabondo				
Arm 1	Reference			
Arm 2	1.93 (0.72–5.13), p = 0.189	10.58 (0.25–450.16), p = 0.218	5.04 (0.92–27.64), p = 0.063	0.13 (0.01–2.01), p = 0.146
Arm 3	2.10 (0.79–5.54), p = 0.135	8.98 (0.20–409.40), p = 0.260	5.98 (1.11–32.11), p = 0.037	0.13 (0.01–2.01), p = 0.146
Arm 4	0.80 (0.25–2.54), p = 0.705	4.62 (0.10–211.94), p = 0.433	1.69 (0.24–12.08), p = 0.602	0.16 (0.01–1.97), p = 0.151
Tolay				
Arm 1	Reference			
Arm 2	0.44 (0.15–1.30), p = 0.136	1.10 (0.15–7.84), p = 0.924	0.25 (0.04–1.61), p = 0.143	0.38 (0.05–3.04), p = 0.361
Arm 3	0.51 (0.18–1.43), p = 0.201	1.15 (0.16–8.04), p = 0.888	0.40 (0.08–1.94), p = 0.256	0.32 (0.04–2.92), p = 0.313
Arm 4	0.29 (0.08–1.05), p = 0.059	0.60 (0.06–6.02), p = 0.664	0.16 (0.01–1.71), p = 0.129	0.32 (0.04–2.92), p = 0.313
*Adult culicine mosquito density*				
Overall				
Arm 1	Reference			
Arm 2	0.69 (0.53–0.89), p = 0.004	0.24 (0.14–0.40), p < 0.001	1.35 (0.85–2.15), p = 0.199	1.03 (0.67–1.60), p = 0.879
Arm 3	0.49 (0.37–0.65), p < 0.001	0.32 (0.20–0.52), p < 0.001	0.52 (0.29–0.92), p = 0.026	0.80 (0.50–1.29), p = 0.366
Arm 4	0.38 (0.28–0.51), p < 0.001	0.30 (0.19–0.47), p < 0.001	0.40 (0.21–0.77), p = 0.006	0.37 (0.21–0.68), p = 0.001
Malindi				
Arm 1	Reference			
Arm 2	0.71 (0.51–0.98), p = 0.037	0.13 (0.06–0.28), p < 0.001	1.60 (0.94–2.71), p = 0.081	1.14 (0.64–2.04), p = 0.652
Arm 3	0.51 (0.36–0.73), p < 0.001	0.26 (0.13–0.49), p < 0.001	0.48 (0.24–0.94), p = 0.032	1.29 (0.67–2.49), p = 0.440
Arm 4	0.22 (0.14–0.35), P < 0.001	0.09 (0.04–0.21), p < 0.001	0.25 (0.11–0.59), p = 0.001	0.65 (0.29–1.45), p = 0.288
Nyabondo				
Arm 1	Reference			
Arm 2	0.85 (0.38–1.89), p = 0.684	0.98 (0.37–2.62), p = 0.973	10.31 (0.02–6705.85), p = 0.480	0.36 (0.06–2.30), p = 0.282
Arm 3	0.55 (0.22–1.37), p = 0.199	0.36 (0.09–1.46), p = 0.153	3.75 (0–3846.25), p = 0.709	0.96 (0.24–3.80), p = 0.949
Arm 4	2.06 (1.06–3.99), p = 0.033	1.73 (0.76–3.95), p = 0.191	37.00 (0.07–19,853.62), p = 0.260	0.64 (0.14–2.96), p = 0.566
Tolay				
Arm 1	Reference			
Arm 2	0.95 (0.40–2.26), p = 0.912	0.68 (0.09–5.01), p = 0.705	0.79 (0.13–4.65), p = 0.795	1.11 (0.35–3.56), p = 0.856
Arm 3	0.79 (0.32–1.95), p = 0.605	0.64 (0.08–4.88), p = 0.667	1.24 (0.25–6.09), p = 0.793	0.62 (0.16–2.36), p = 0.484
Arm 4	0.67 (0.26–1.76), p = 0.420	1.24 (0.22–6.94), p = 0.807	0.90 (0.15–5.33), p = 0.911	0.32 (0.06–1.66), p = 0.174

DR: Density ratio was calculated using generalized estimating equations taking into account the study randomization, it was used to show the intervention effect on adult vector density

Arm 1 referred to LLINs only; Arm 2 combination of LLINs and *Bti*; Arm 3 combination of LLINs and CEM; and Arm 4 combination of LLINs, *Bti*, and CEM

Mosquito larvae were collected in different habitats around the sampled houses in all the 16 villages in the three study sites. Characteristics of potential aquatic breeding habitats generally fell in the following three categories based on their estimated lifespan: temporary habitats lasting from 2 weeks up to about 3 months including rain puddles, shallow drainage pools especially by the sides of dirt roads, shallow soil excavation sites left behind after brick making activities, and car tracks; semi-permanent habitats lasting up to 6 months including ground pools, seepage pools next to streams, brick-making pits, shallow ponds by the edge of streams; and, permanent habitats lasting more than 6 months including ponds used for domestic water and watering of livestock, fish ponds, wells. The combined semi-permanent and permanent habitats in a village in either Nyabondo or Malindi were about twice as many as those in a village in Tolay.

**Fig. 4:**
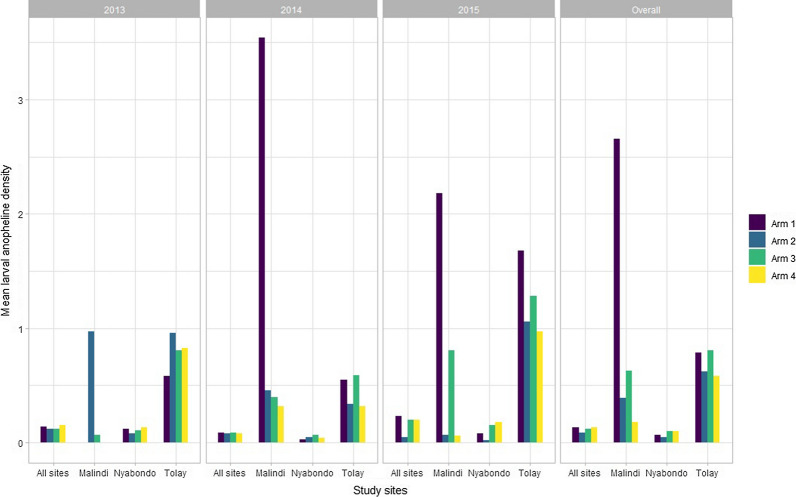
Mean larval mosquito densities at different survey years in Malindi, Nyabondo and Tolay sites

The overall larval anopheline density was 0.12 with highest anopheline larval density observed in Tolay site (0.66) followed by Malindi (0.55) and Nyabondo (0.08) (Fig. 4). Similarly, the overall larval culicines density was 0.18 with the highest larval culicines density observed in Tolay (1.20), followed by Malindi (0.42) and Nyabondo (0.13) sites. In overall, arm 2 had the least larval vector density for both anophelines and culicines compared to other study arms. At individual site level, arm 2 had the least larval density for anophelines and culicines in Nyabondo and Tolay study sites.

Analysis of the effect of the treatment interventions on larval vector density showed that in overall arm 2 of the study significantly reduced the larval anophelines density by nearly a third (DR = 0.65, p < 0.001), and also significantly reduced the larval culicines density by nearly 12% (DR = 0.88, p = 0.0016). In two study sites, Malindi and Nyabondo, arm 2 significantly reduced the larval anophelines density by 85 and 20% in the two sites, respectively. However, the larval culicines density was significantly reduced in arm 2 and both arms 2 and 3 only in Nyabondo and Malindi study sites, respectively (Table 3).

**Table 3:** Comparison of the effects of the treatment interventions on larval mosquito density in the sampled villages in Malindi, Nyabondo and Tolay study sites using generalized estimating equations

Interventions	Overall	Year 1: 2013	Year 2: 2014	Year 3: 2015
	DR, p-value	DR, p-value	DR, p-value	DR, p-value
*Anopheline larval mosquito density*				
Overall				
Arm 1	Reference			
Arm 2	0.65 (0.57–0.74), p < 0.001	0.90 (0.73–1.11), p = 0.330	0.85 (0.70–1.04), p = 0.124	0.21 (0.16–0.28), p < 0.001
Arm 3	0.93 (0.83–1.05), p = 0.234	0.85 (0.69–1.06), p = 0.143	0.92 (0.76–1.13), p = 0.447	0.88 (0.72–1.08), p = 0.225
Arm 4	0.96 (0.85–1.07), p = 0.441	1.11 (0.90–1.36), p = 0.340	0.83 (0.68–1.02), p = 0.072	0.85 (0.69–1.05), p = 0.125
Malindi				
Arm 1	Reference			
Arm 2	0.15 (0.10–0.21), p < 0.001	39.74 (0.01–311,676.8), p = 0.421	0.13 (0.08–0.21), p < 0.001	0.03 (0.01–0.08), p < 0.001
Arm 3	0.24 (0.16–0.36), p < 0.001	2.72 (0–40,914.24), p = 0.839	0.11 (0.05–0.24), p < 0.001	0.37 (0.22–0.62), p < 0.001
Arm 4	0.07 (0.05–0.10), p < 0.001	0	0.09 (0.05–0.15), p < 0.001	0.03 (0.01–0.06), p < 0.001
Nyabondo				
Arm 1	Reference			
Arm 2	0.80 (0.67–0.94), p = 0.008	0.68 (0.54–0.87), p = 0.002	1.44 (1.06–1.94), p = 0.018	0.26 (0.16–0.41), p < 0.001
Arm 3	1.54 (1.32–1.79), p < 0.001	0.87 (0.69–1.09), p = 0.215	2.17 (1.63–2.88), p < 0.001	1.99 (1.47–2.69), p < 0.001
Arm 4	1.51 (1.29–1.76), p < 0.001	1.05 (0.84–1.32), p = 0.675	1.35 (0.99–1.84), p = 0.058	2.33 (1.72–3.15), p < 0.001
Tolay				
Arm 1	Reference			
Arm 2	0.79 (0.55–1.11), p = 0.175	1.65 (0.86–3.16), p = 0.134	0.62 (0.35–1.10), p = 0.101	0.63 (0.32–1.24), p = 0.182
Arm 3	1.03 (0.69–1.52), p = 0.892	1.39 (0.67–2.89), p = 0.370	1.07 (0.57–2.02), p = 0.840	0.76 (0.36–1.60), p = 0.468
Arm 4	0.74 (0.53–1.03), p = 0.077	1.42 (0.75–2.67), p = 0.277	0.59 (0.34–1.02), p = 0.059	0.58 (0.31–1.08), p = 0.086
*Culicine larval mosquito density*				
Overall				
Arm 1	Reference			
Arm 2	0.88 (0.79–0.98), p = 0.016	0.59 (0.50–0.71), p < 0.001	2.46 (2.01–3.02), p < 0.001	0.39 (0.32–0.48), p < 0.001
Arm 3	1.23 (1.11–1.37), p < 0.001	1.06 (0.90–1.25), p = 0.489	1.84 (1.48–2.28), p < 0.001	0.86 (0.72–1.04), p = 0.114
Arm 4	1.37 (1.24–1.52), p < 0.001	1.05 (0.89–1.25), p = 0.535	2.27 (1.85–2.79), p < 0.001	1.11 (0.93–1.32), p = 0.244
Malindi				
Arm 1	Reference			
Arm 2	3.33 (1.93–5.76), p < 0.001	13.41 (0–41,102.22), p = 0.526	12.44 (2.55–60.58), p = 0.002	2.81 (1.49–5.27), p = 0.001
Arm 3	1.82 (0.96–3.45), p = 0.064	20.09 (0.01–68,418.63), p = 0.470	2.79 (0.45–17.39), p = 0.272	1.53 (0.75–3.13), p = 0.246
Arm 4	1.04 (0.59–1.84), p = 0.880	0	6.37 (1.31–31.12), p = 0.022	0.41 (0.20–0.83), p = 0.014
Nyabondo				
Arm 1	Reference			
Arm 2	0.65 (0.57–0.75), p < 0.001	0.57 (0.47–0.70), p < 0.001	2.88 (2.13–3.89), p < 0.001	0.10 (0.07–0.15), p < 0.001
Arm 3	1.51 (1.34–1.70), p < 0.001	1.19 (1.00–1.42), p = 0.052	3.49 (2.60–4.70), p < 0.001	0.97 (0.79–1.19), p = 0.759
Arm 4	1.41 (1.25–1.59), p < 0.001	1.10 (0.92–1.32), p = 0.310	2.09 (1.53–2.87), p < 0.001	1.32 (1.08–1.61), p = 0.006
Tolay				
Arm 1	Reference			
Arm 2	0.69 (0.51–0.93), p = 0.014	0.72 (0.41–1.28), p = 0.269	0.76 (0.49–1.16), p = 0.203	0.56 (0.30–1.07), p = 0.078
Arm 3	0.64 (0.45–0.91), p = 0.014	0.84 (0.45–1.56), p = 0.576	0.64 (0.38–1.10), p = 0.106	0.45 (0.21–0.94), p = 0.035
Arm 4	0.78 (0.59–1.03), p = 0.082	0.83 (0.49–1.42), p = 0.500	0.88 (0.58–1.33), p = 0.537	0.57 (0.32–1.03), p = 0.064

DR: Density ratio was calculated using generalized estimating equations taking into account the study randomization, it was used to show the intervention effect on larval vector density

Arm 1 referred to LLINs only; Arm 2 combination of LLINs and *Bti*; Arm 3 combination of LLINs and CEM; and Arm 4 combination of LLINs, *Bti*, and CEM

### Epidemiological findings from the school-level surveys

Baseline demographic and epidemiological characteristics for the school children is shown in Table 1. A total of 3599 children (38 schools) with median age of 9 years (range: 3–19 years) were included in the baseline cross-sectional survey in all the three sites. The reported household membership was five people per household with high household membership observed in Malindi study site. Baseline overall malaria prevalence was low 8.8% (95% CI 5.5–14.0%) with the baseline prevalence being significantly different in each of the four study arms, χ^2^ = 216.06, p < 0.001 and ranged from 5.1 to 12.6%.

Table 4 outlines the number of schools and children surveyed, malaria prevalence and the overall treatment interventions effect on malaria prevalence. The overall malaria prevalence was 13.3% (95% CI 8.8–20.0%) with the highest malaria prevalence observed in Nyabondo site 32.4% followed by Malindi 5.7% and Tolay 1.7%. In overall, highest malaria prevalence was observed in the third year of the survey (the year 2015) compared to the other 2 years.

**Table 4:** Effect of the four treatment interventions on *Pf*PR and adjusted for risk factors based on the school surveys in Malindi, Nyabondo and Tolay study sites

	Schools (children)	Year 1: 2013	Year 2: 2014	Year 3: 2015	Overall	
		*Pf*PR % [95% CI]^b^	*Pf*PR % [95% CI]^b^	*Pf*PR % [95% CI]^b^	*Pf*PR % [95% CI]^b^	Multivariable^a^ aOR [95% CI], p-value
Overall	38 (11,846)	5.4 (2.4–12.1)	6.9 (4.4–10.7)	23.9 (16.2–35.1)	13.3 (8.8–20.0)	
Malindi	10 (3000)	5.4 (2.4–12.1)	7.9 (4.4–14.2)	3.7 (1.9–7.2)	5.7 (3.1–10.4)	Reference
Nyabondo	12 (4046)	–	15.7 (10.9–22.6)	49.5 (41.5–58.9)	32.4 (26.1–40.1)	9.1 (7.6–10.9), p < 0.001
Tolay	16 (4800)	–	1.0 (0.7–1.4)	3.2 (2.5–4.1)	1.7 (1.4–2.1)	0.3 (0.2–0.4), p < 0.001
*Study arm*						
Overall						
Arm 1	19 (3240)	1.6 (0.6–4.0)	6.1 (3.0–12.3)	13.0 (5.6–30.3)	7.7 (3.8–15.5)	Reference
Arm 2	18 (2942)	12.0 (4.4–32.6)	9.3 (4.6–18.6)	28.1 (15.6–50.5)	17.1 (9.5–30.7)	2.1 (1.7–2.5), p < 0.001
Arm 3	15 (2193)	4.0 (3.8–4.2)	4.1 (1.8–9.1)	19.6 (8.8–44.0)	10.4 (4.3–24.9)	0.9 (0.7–1.1), p = 0.356
Arm 4	16 (2643)	6.0 (5.6–6.3)	5.8 (2.8–11.8)	35.2 (21.1–58.7)	19.3 (10.3–36.1)	1.7 (1.4–2.0), p < 0.001
Malindi						
Arm 1	5 (1500)	1.6 (0.6–4.0)	5.8 (1.7–20.2)	2.0 (1.0–4.0)	3.1 (1.1–8.9)	Reference
Arm 2	3 (900)	12.0 (4.4–32.6)	13.3 (7.7–23.0)	7.7 (3.3–17.7)	11.0 (5.4–22.5)	3.8 (2.7–5.5), p < 0.001
Arm 3	1 (300)	4.0 (3.8–4.2)	2.0 (1.8–2.2)	1.0 (0.8–1.2)	2.3 (2.0–2.6)	0.7 (0.3–1.7), p = 0.460
Arm 4	1 (300)	6.0 (5.6–6.3)	8.0 (7.6–8.2)	3.0 (2.4–3.4)	5.7 (5.2–6.0)	1.9 (1.1–3.3), p = 0.033
Nyabondo						
Arm 1	2 (400)	–	18.5 (9.7–35.0)	47.3 (35.8–62.3)	31.1 (21.2–45.6)	Reference
Arm 2	4 (1380)	–	24.5 (18.2–33.0)	61.6 (46.0–82.4)	45.6 (37.0–56.2)	1.9 (1.5–2.3), p < 0.001
Arm 3	2 (816)	–	14.0 (10.3–18.9)	40.8 (28.1–59.3)	29.1 (20.3–41.8)	0.9 (0.7–1.2), p = 0.456
Arm 4	4 (1450)	–	14.6 (9.9–21.5)	58.4 (46.9–72.7)	41.8 (35.6–49.1)	1.6 (1.3–2.0), p < 0.001
Tolay						
Arm 1	4 (1200)	–	1.6 (1.2–2.2)	4.3 (3.7–5.0)	2.4 (1.9–2.9)	Reference
Arm 2	4 (1200)	–	0.9 (0.4–2.0)	3.3 (1.5–6.9)	1.7 (1.0–2.9)	0.7 (0.4–1.3), p = 0.235
Arm 3	4 (1200)	–	0.6 (0.2–1.7)	3.5 (3.0–4.1)	1.6 (1.2–2.2)	0.6 (0.4–1.2), p = 0.180
Arm 4	4 (1200)	–	0.8 (0.3–1.7)	2.0 (1.0–4.0)	1.2 (1.0–1.4)	0.5 (0.3–0.9), p = 0.032
Age (years)						
≤ 5	739 (6.3%)	2.6 (0.2–30.6)	7.1 (3.9–13.0)	52.3 (39.2–69.8)	36.0 (26.1–49.7)	2.3 (1.8–2.8), p < 0.001
6–10	6787 (57.8%)	5.5 (2.4–12.6)	9.4 (6.4–14.0)	26.0 (17.5–38.6)	15.5 (10.5–22.8)	1.6 (1.3–1.9), p < 0.001
> 10	4216 (35.9%)	6.7 (1.3–33.9)	4.1 (2.3–7.2)	9.4 (5.4–16.3)	5.8 (3.4–9.9)	Reference
Gender						
Male	6021 (51.4%)	6.1 (2.6–14.1)	7.5 (4.7–12.0)	23.1 (15.7–34.0)	13.5 (9.0–20.4)	1.1 (0.9–1.2), p = 0.635
Female	5704 (48.7%)	4.7 (1.9–11.5)	6.3 (4.2–9.7)	24.7 (16.7–36.5)	13.0 (8.6–19.8)	Reference

^a^Effects of interventions on malaria prevalence was analyzed using logistic regression model

^b^Malaria prevalence was analyzed using binomial logistic regression model taking into account school clusters

–: No data collection took place at those time points

Arm 1 referred to LLINs only; Arm 2 combination of LLINs and *Bti*; Arm 3 combination of LLINs and CEM; and Arm 4 combination of LLINs, *Bti*, and CEM

Overall, the least malaria prevalence was observed in the control arm (7.7%) and highest prevalence in arm 4 of the study (19.3%). However, arm 3 of the study showed least prevalence in two study sites, Malindi and Nyabondo, with arm 4 showing the least prevalence in Tolay site only.

**Fig. 5:**
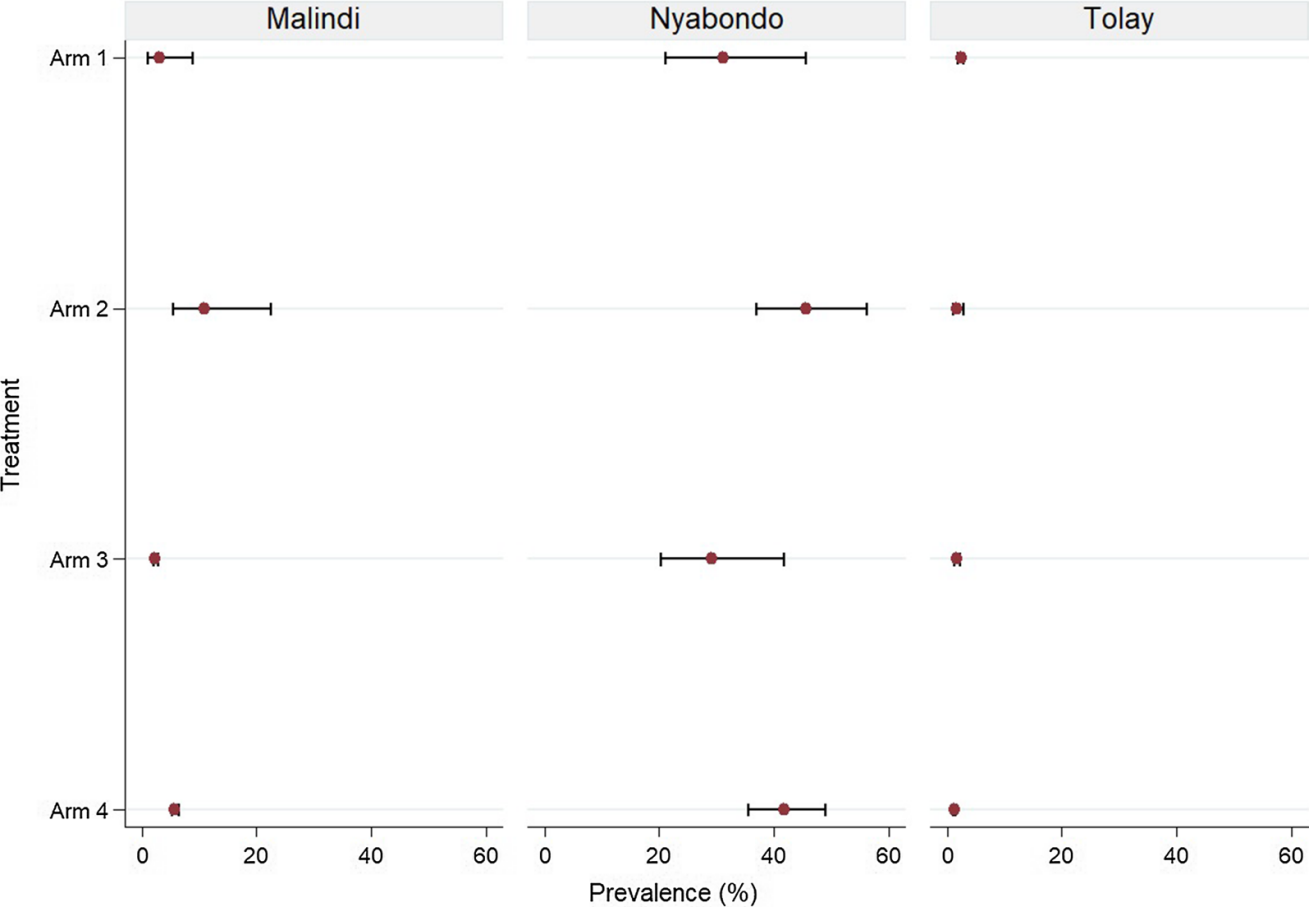
*Plasmodium falciparum* prevalence (*Pf*PR) in Malindi, Nyabondo and Tolay sites from 2013 to 2015

Figure 5 provides the site-specific malaria prevalence by the different treatment interventions. At global level, analysis of the effect of the treatment interventions on malaria prevalence showed that only arm 3 reduced malaria prevalence by 10% (aOR = 0.9, p = 0.356), although this was not significant. The other intervention combinations did not show any prevalence reduction when compared to the control arm of the study. However, at individual site level Tolay showed significant reduction in malaria prevalence in arm 4 by 50% (aOR = 0.5, p = 0.032), while Malindi and Nyabondo showed no prevalence reduction in any of the study arms.

The results demonstrated that children under 5 years of age (aOR = 2.3, p < 0.001) as well as those aged 6 to 10 years (aOR = 1.6, p < 0.001) were at significant risk of malaria infection when compared to older children (over 10 years). However, male children were at non-significant risk of malaria infection compared to females, aOR = 1.1, p = 0.635.

## Discussion

The study assessed the impact of integrating the usage of LLINs with larviciding with *Bti,* and intensive CEM, on malaria vector populations and malaria prevalence at three rural sites, namely Nyabondo and Malindi in Kenya, and Tolay in Ethiopia. While LLINs and *Bti* are the actual mosquito control methods, CEM is a social intervention which is necessary for their implementation and sustainability. By analysing the individual and combined effects of two technical and one social interventions, the study aimed at a better understanding of their respective contributions in the set-up broadly defined as IVM strategy [12, 13, 45]. This is the first time in the study areas when a direct comparison of such functionally different technical and social interventions has been attempted, to evaluate their individual and synergistic effects as key elements of IVM.

The relative density of anopheline mosquitoes indoors was found to be generally very low at all the three sites, being less than one mosquito per house per night. The overall average malaria prevalence varied considerably across the three sites with the order of magnitude being 19:3:1 for Nyabondo:Malindi:Tolay, respectively. The assessment of the adult anopheline density at the combined three-site (global level) showed that compared to the control arm (use of LLINs only), LLINs coupled with *Bti* application and CEM (arm 4) significantly reduced by half the adult anopheline density. Nonetheless, at individual site level, no significant reduction in adult anopheline density was observed in any of the three sites.

For the adult culicines density, the global analysis revealed that all three study arms (i.e., arms 2, 3 and 4) were significantly effective in reducing adult culicines density, with arm 4 showing up to two-thirds reduction. At site-specific level, two sites (i.e., Nyabondo and Tolay) did not show significant reduction of adult culicines density in any of the interventions, however, Malindi site showed that arms 3 and 4 were significantly effective in reducing adult culicines density.

Similarly, assessment of larval anopheline density at global level revealed that arm 2 was significantly effective in reducing larval anopheline density by up to a third. At site-specific level, all the study arms showed significant reduction of larval anopheline density in Malindi by at least 70%, while in Nyabondo only arm 2 reduced density by 20%, and no study arm showed significant reduction of larval anopheline density in Tolay.

Study arm 2 was similarly effective in reducing the overall larval culicines density by about 12%, with the same study arm reducing larval culicines density by nearly a third in Nyabondo site. In Tolay site, both arms 2 and 3 were significantly effective in reducing larval culicines density by at least 30%. However, no significant reductions were observed in any of the interventions in Malindi site.

At global level, arm 3 reduced malaria prevalence by *circa* 10% though this was not statistically significant. However, it is important to point out that in Tolay site, malaria prevalence was significantly reduced by 50% in arm 4. The two other sites did not reveal significant reduction of prevalence as a result of the use of any of the interventions.

The results of parasite prevalence obtained during this study were similar to what has previously been reported for the three respective project sites [3, 4]. In this regard, the results confirmed that Tolay and Malindi had significantly lower malaria prevalence than Nyabondo as presumed during the selection of the study sites. The prevalence results at the three project sites importantly favoured the original intention of the study, i.e., to have the IVM interventions assessed among situations with low and moderate to high malaria prevalence. In this regard, it was interesting to note that compared to the control (use of LLINs only), the adopted relatively comprehensive strategy in arm 4 significantly reduced malaria prevalence in the setting with the lowest malaria prevalence, i.e., Tolay. The same strategy reduced the relative densities of anopheline and culicine mosquitoes in the settings with higher malaria prevalence. However, in these latter settings neither the relatively comprehensive intervention strategy, nor the strategies separately having either CEM or *Bti*, had a significant reduction of malaria prevalence when compared to the control arm.

A logical inference based on the above evidence is that bringing on board larviciding with *Bti* can help to reduce malaria further in settings where prevalence is already low, either naturally or due to conventional implementation of a primary vector control method such as LLINs. As regards the study sites with moderate to high malaria prevalence and more extensive potential anopheline breeding habitats, the results of *Bti* application corroborated previous assertions of low or no additional impact of larviciding in malaria control, as expressed in a recent systematic review of past studies on the intervention [46].

According to the results, low adult anopheline densities recorded at all three sites during the 2013–2015 period of the study did not necessarily translate to equally low malaria prevalence across the sites. Thus, malaria prevalence in Nyabondo and Malindi sites in Kenya remained higher than in Tolay, in spite of adult anopheline densities being similarly low at less than one mosquito collected per trap per night indoors. Interestingly, this situation contrasts with that in a follow-up study from 2017 to 2018 in Nyabondo alone, where indoor anopheline densities were higher, and screening of house eaves was found to lower indoor anopheline density as well as malaria parasite prevalence [27]. Further research may therefore be needed to understand how mosquito relative density and other entomological variables of the vectorial capacity [47] of anopheline populations in an area might vary over time, and the implications of any such variation for malaria prevalence in the human population. Other factors which may require further investigation as part of understanding malaria dynamics in an area include certain human behaviour, such as sleeping, working or socializing outdoors at night, which are associated with outdoor malaria transmission [48].

In Malindi, the study demonstrated significantly lower anopheline and culicine larval populations in the experimental arms that had *Bti*, i.e., arm 2 and arm 4, compared to those that did not have *Bti*. The results support previous findings of *Bti’s* known high efficacy against mosquito larvae under controlled conditions [49], However, the failure to also significantly reduce adult anopheline mosquitoes at the same site in the same study arms involving the application of *Bti* likely point to operational gaps in the way larviciding was conducted. Thus, some of the potential anopheline breeding sites might have gone untreated due to failure to detect them, especially during the wet season when they are many and spread over a wide area. WHO policy recommendation in response to this particular challenge as it relates to malaria vector control is that larviciding should only be considered, with or without other interventions, when the breeding sites in the targeted area are few, fixed and findable [50]. Consequently, WHO recommends that prior to adding more interventions in an actual vector control situation, an assessment should be made to determine if the ineffectiveness of what is currently being applied is due to operational shortcomings that may require resolving through improved management rather than adding of new vector control methods. Operational problems may arise due to inadequate human resources, poor supervision, or lack of tools for mapping and tracking mosquito habitats.

In the case of Tolay study site in Ethiopia, it should be noted that vector control also often involves the use of IRS in addition to LLINs, and this could generally have also contributed to low indoor anopheline vector densities observed in the study site [4, 51]. However, combining LLINs with IRS has been shown not to further reduce malaria incidence beyond what is achieved with LLINs in Ethiopia in spite of its having an impact on indoor vector density [52]. Finally, low indoor anopheline densities in Tolay could also have been due to local houses not having open eaves. On the other hand, low densities in Nyabondo and Malindi could have been due to other environmental, socio-economic or past vector control factors since there was no IRS at the two sites during the study period, and house eaves were generally open.

CEM in the study was expected to influence communities’ behaviour leading to a range of anti-vector activities, including improved usage of LLINs and general environmental management. The lack of significant additional impact on adult anopheline density and malaria prevalence in the experimental arm where CEM was added to LLINs could suggest that, in spite of household net ownership being relatively high and meeting universal coverage, net usage, although not monitored during the study, might have been below the desired optimal levels anticipated in universal coverage [29]. Results on the assessment of how CEM changes people’s knowledge and practice towards vector control will be published separately. However, such an assessment conducted in other research in Tolay area of Ethiopia has recently confirmed that malaria education conveyed through primary school pupils significantly improves community knowledge and leads to behaviour change towards malaria and malaria vector control [53].

## Limitations

Certain challenges were encountered in the study methodology which require that the results are interpreted with caution, but which may also provide a useful basis for refinement of future similar studies. For instance, larviciding with *Bti* and CEM were interventions that essentially operated at community level, while the usage of LLINs is a self-protection measure at personal and household level, in spite of having a communal dimension when scaled up in a village setting. Thus, implementing the former two interventions could potentially have led to unintended spillover effects among the study clusters in spite of randomization efforts. For instance, a school enrolled in the cross-sectional malaria surveys and associated with a particular IVM intervention might have had some of its pupils who were tested for malaria resident in clusters with different interventions, but not recorded as such. Furthermore, the CEM intervention may have been confounded by intermingling of residents of different clusters. The problem of spillover effects is well known and has been a subject of discussion in other studies on educational interventions [54]. Moreover, it is also likely that certain information about mosquito and malaria control was generally available at all the study sites due to their being located in areas where malaria research and other anti-malaria activities have been going on for many years, especially in Kenya [19, 55–57].

Furthermore, information on universal net coverage was not verified through actual household surveys. Nevertheless, congruence of data from other detailed studies and MIS reports lends credibility to net coverage as cited for the different study sites in “Methods” section of this paper. Lack of more detailed information about larval breeding sites and unavailability of current insecticide resistance test data for the respective study sites were also important limitations of the study.

## Conclusions

The study demonstrated that integration of conventional usage of LLINs with larviciding with *Bti* and CEM led to further reduction in malaria in a setting of low disease prevalence in Tolay in Ethiopia, but not in settings with relatively higher prevalence in Kenya. The results suggest that reducing malaria beyond what has been achieved with LLINs in the latter settings would require a different IVM configuration, possibly including other anti-vector measures as well as integration with prompt diagnosis and effective treatment of malaria cases. The observations underscore the need to view IVM as an adaptive approach which may need to be calibrated differently for different settings based on prior knowledge of the socio-ecological situation, disease epidemiology and effectiveness and practicality of interventions as is often reiterated by WHO [58].

## Data Availability

The data supporting the conclusions of this article are provided within the article. The datasets analyzed are available upon request.

## Abbreviations

ACT: Artemisinin-based combination therapy
aOR: Adjusted odds ratio
*Bti*: *Bacillus thuringiensis israelensis*
*CEM*: Community education and mobilization
CDC: Centers for Disease Control
CI: Confidence interval
DR: Density ratio
GEE: Generalized estimating equations
IEC: Information, education and communication
IRRs: Incidence rate ratios
IRS: Indoor residual spraying
IVM: Integrated vector management
LLINs: Long-lasting insecticide treated nets
MOH: Ministry of Health
NMCPs: National Malaria Control Programmes
PCR: Polymerase chain reaction
*Pf*PR: *Plasmodium falciparum* prevalence
RDT: Rapid diagnostic test
SEs: Standard errors
WHA: World Health Assembly
WHO: World Health Organization
